# Oral health status and cardiovascular risk profile in Cameroonian military population

**DOI:** 10.3934/publichealth.2021008

**Published:** 2021-01-13

**Authors:** W Bell Ngan, L Essama Eno Belinga, SAP Essam Nlo'o, E Abeng Mbozo'o, E Otsomoti, J Mekoulou Ndongo, EC Bika Lele, D Hupin, SH Mandengue, F Roche, B Bongue

**Affiliations:** 1 Department of Military Health, Yaoundé, Cameroon; 2 Physical Activities and Sport Physiology and Medicine Unit, Faculty of Science, University of Douala, Cameroon; 3 INSERM, U1059, Sainbiose, Dysfonction Vasculaire et Hémostase, Université de Lyon, Université Jean Monnet, Saint-Etienne, France; 4 Douala General Hospital, Cameroon; 5 Faculty of Medicine and Pharmaceutical Sciences, University of Douala, Cameroon; 6 Fifth Military Sector Health Center, Ngaoundéré, Cameroon; 7 Cetaf, St-Etienne, France

**Keywords:** oral health, cardiovascular risk, cardiovascular diseases, militaries

## Abstract

**Background:**

Periodontal diseases (PD) seem to appear today as predictors of some cardiovascular diseases (CVD). There is a lack of data on the oral health among Cameroonian military population, and its relationship with CVD.

**Purpose:**

Investigate on the link between oral health of Cameroonian military from the Ngaoundéré garrison and their cardiovascular risk profile.

**Participants and methods:**

A cross-sectional study at the Fifth Military Sector Health Center in Ngaoundéré was conducted. General health parameters assessment was done according to the World Health Organization STEPS manual for surveillance of risk factors for non-communicable chronic diseases and the Alcohol Use Disorders Identification Test. The periodontal status was assessed using Dutch Periodontal Screening Index.

**Results:**

Two hundred and five participants who were officers and non-commissioned officers (aged 47 ± 08 and 32 ± 08 years respectively), with 86.4% of men were included. Smoking was associated to periodontitis (OR = 4.44 [1.73–11.43], p = 0.0031). Quality of oral hygiene was associated to high cardiovascular risk profile, poor/good (OR = 3.96 [1.07–14.57], p = 0.0386) and medium/good (OR = 3.44 [1.11–10.66], p = 0.0322).

**Conclusion:**

Lifestyle as tobacco consumption and poor oral hygiene were associated to CVD among military, and this call for change.

## 1. Introduction

Noncommunicable diseases (NCDs), also known as chronic diseases, are one of the major challenges for health and sustainable human development. To date, the global NCD response has focused on the four major NCDs: cardiovascular (heart), diabetes, chronic respiratory diseases, cancers [1]–[3]. The association between oral health status and cardiovascular health has been highlighted by numerous studies [4]–[9]. Indeed, some oral diseases such as periodontal diseases (PD) seem to appear today as predictors of cardiovascular diseases (CVD) [7]. Blaizot et al. [5] found that patients with PD, the risk of developing CVD is doubled. Similarly, evidence on the role of periodontitis in the genesis of atherosclerosis is reported in several publications [10]–[12]. Periodontal diseases being inflammatory pathologies, they induce the production of inflammatory mediators such as tumor necrosis factor α (TNF-α), interleukins (IL-1, IL-6, IL-8) and the C-reactive protein (CRP) that have been shown to be associated with atherogenesis [10]–[12]. In addition, certain bacteria of the oral flora such as *Porphyromonas gingivalis (Pg)*, *Aggregatibacter actinomycetemcomitans (Aa)* and *Streptococcus sanguis (Ss)* have been found in patch of atheroma probably due to transient bacteremia by crossing the dental sulcus [10]–[12]. Finally, more recent work demonstrates the beneficial effect of periodontal therapeutics on endothelial function [13].

CVD (ischemic heart disease, stroke, peripheral vascular disease) are responsible for a third of global world mortality, i.e. 17.9 million deaths worldwide and 147.6 thousand deaths in sub-Saharan central Africa [14]. The fight against the spread of CVD requires the reduction and control of classical cardiovascular risk factors such as smoking, harmful alcohol consumption, physical inactivity, unbalanced diet, high blood pressure, hypercholesteromia, diabetes, and obesity among others, but also the reduction of poor oral health [15],[16]. To achieve this, the level of exposure of the populations should first be identified.

The military population did not seem to be exposed to CVD, yet published studies on this topic showed that military personnel are equally exposed to CVD as civilians and sometimes even more [16],[17]. A recent meta-analysis on military population reveals that frequencies of cardiovascular risk factors among soldiers were 26% for hypertension, 9% for hyperglycemia, 14% for obesity, and 35% for overweight [16]. Another study comparing military personnel to civilians found significant associations between being military personnel and smoking; being military personnel and excessive alcohol consumption; and being veteran and CVD [17].

Military personnel are generally required to work for long hours, or deployed for fighting operations, which exposes them to stress, fatigue, excessive consumption of tobacco and/or alcohol, less body and oral hygiene time. All of these factors contribute to increase susceptibility of military personnel for oral diseases including PD [18]–[22]. A meta-analysis, focusing on soldiers deployed in fighting areas, soldiers in maneuvers, soldiers in submarine missions and soldiers in mission in Antarctica, reveals that among the oral pathologies encountered, the frequencies of PD were 6.4%, 8.6%, 21.6% and 10.0% respectively [23].

Numerous works have been dedicated to the distribution of CVD and cardiovascular risk factors in Cameroon.

However, to our knowledge, there is no data on the oral health of Cameroonian soldiers, even less on the relationship that could exist between this oral health and the general health of the Cameroonian military population. Thus, the objective of this study was to assess the link between oral health of soldiers from the Ngaoundéré garrison and their cardiovascular risk profile.

## 2. Methods

### 2.1. Study design

This was a descriptive and analytical cross-sectional study.

### 2.2. Study area and population

The study concerned soldiers of the garrison of Ngaoundéré, a city located in the Adamawa region of Cameroon and headquarter of Sector N°5. Were included in the present study, any soldier who attended the Military Medical Center of Sector N°5 for the annual re-engagement medical visit and accepted to be submitted to an oral examination in addition to the general systematic clinical examination. Those with any diagnosed cardiovascular disease was excluded.

### 2.3. Medical examinations, data collected and definitions of variables

A questionnaire developed from the World Health Organization (WHO) STEPS manual for surveillance of risk factors of NCDs was used to collect socio-demographic information (age, gender, military rank); information on habits related to healthy living, alcohol consumption and smoking (answering by “yes” or “no” to the question) in particular; and information about the medical history of study participants. An additional tool, the Alcohol Use Disorders Identification Test (AUDIT) was used to assess the participant's level of alcohol consumption. Participant's anthropometric data (height and weight) were measured to determine their Body Mass Index (BMI).

Each participant's blood pressure was measured using an electronic blood pressure monitor (BP-103H, IDASS, Unit W2-3, Scarne Business Park, Launceston, Cornwall, United-Kingdom), at a seated position after resting for 15min. Measurements were made on each arm after with ten minutes between two measurements. When participants diastolic and/or systolic pressures ≥90 mmHg and/or 140 mmHg respectively, a second measurement was made on both arms after ten minutes. In all cases, the lowest values ​​were considered for each participant. Blood pressure superior to normal (BPSN) was considered when systolic blood pressure ≥140 mmHg and/or diastolic ≥90 mmHg.

The fasting capillary blood glucose test was carried out using strips and glucometer (One Touch Ultra®2, Lifescan Canada Ltd, 675 Avenue Belvédère, Quebec, QC G1S 3E6, Canada). Fasting blood glucose ≥7 mmol/L was considered diabetic.

Full-mouth examination was carried using the Oral hygiene index (OHI); the Decayed, Missing, and Filled teeth Index (DMFT) for the level of tooth decay; and the Dutch Periodontal Screening Index (DPSI) for periodontal status.

a) Quality of oral hygiene: 3 modalities were defined according to OHI. Poor (OHI >3), medium (1.3≤ OHI <3), good (OHI ≤1.2).

b) Healthy periodontium: DPSI = 0.

c) Gingivitis: DPSI = 1 or 2.

d) Periodontitis: DPSI ≥3.

The cardiovascular risk was evaluated by summing the participant's cardiovascular risk factors, precisely: smoking, excessive alcohol consumption (defined by an AUDIT score ≥8), overweight or obesity, hypertension, and diabetes. The cardiovascular risk was considered high if the participant had a combination of at least 3 risk factors.

### 2.4. Statistical analysis

All analyzes were performed using Epi info version 7.2 software (CDC, Atlanta, Georgia). The Fisher exact test was used for comparing proportions. The Mann-Whitney test and the analysis of variances (ANOVA) were carried out according to their indications to compare numeric variables. Univariate and multivariate logistic regressions were performed to analyze the relationship between periodontal parameters and high cardiovascular risk. The significance threshold was set at p ˂ 0.05.

### 2.5. Ethical considerations

The study was authorized by the military hierarchy (ref. 016082/AU/DSM/RSM3/SSM5) and the participants all agreed to the use of the information collected for the purposes of this study. The study was conducted in conformity with the recommendations of the Declaration of Helsinki revised in 1989.

## 3. Results

### 3.1. General characteristics of the sample

Two hundred and five participants were included, among whom 188 (91.7%) were non-commissioned officers (NCOs). The participant's age range was 22 to 58 years. Officers were older than NCOs (47 ± 08 versus 32 ± 08 years; p = 0.00001). Males represented 86.4% of the sample. 182 (88.8%) participants were non-smokers. 142 (69.2%) participants included participants consumed alcohol. The proportion of participants consuming alcohol was higher among officers than among NCOs (94.1% (16) versus 67.0% (126); p = 0.0251).

Concerning weight status, 80 (39.5%) participants were normal weight, 01 (0.5%) was underweight, 100 (48.8%) were overweight and 23 (11.2%) obese.

The number of cardiovascular risk factor per subject was higher among officers than the NCOs (medians [inter quartile range]: 2 [2–3] versus 1 [1–2]; p = 0.0032).

**Table 1.** General characteristics of the sample.

Characteristics	Total (n = 205)	Officers (n = 17)	NCOs (n = 188)	p*
	Count (%)			
Gender				
Women	28 (13.66)	2 (11.76)	26 (13.83)	1.0000
Men	177 (86.34)	15 (88.24)	162 (86.97)	
Smoking	23 (11.22)	2 (11.76)	21 (11.17)	1.0000
Excessive alcohol consumption	122 (59.51)	13 (76.47)	109 (57.98)	0.1970
Obesity or overweight	123 (60.00)	14 (82.35)	109 (57.98)	0.0688
Hypertension	33 (16.10)	6 (35.29)	27 (14.36)	0.0364
Diabetes	3 (1.46)	1 (5.88)	2 (1.06)	NA
High cardiovascular risk	29 (14.15)	6 (35.29)	23 (12.23)	0.0192
Gingivitis	130 (64.41)	7 (41.18)	123 (65.43)	0.0644
Periodontitis	32 (15.61)	3 (17.65)	29 (15.43)	0.7334
	Median [IQR]			p**
Age (years)	28 [26–39]	48 [43–53]	28 [26–36]	0.0000
SBP (mmHg)	125 [116–135]	134 [124–143]	125 [116–135]	0.0152
DBP (mmHg)	78 [69–85]	82 [78–89]	77 [68–84]	0.0081
BMI (Kg/m^2^)	25.8 [23.8–27.8]	27.8 [25.8–29.4]	25.6 [23.7–27.5]	0.0138
Glycemia (mmol/l)	5.19 [4.88–5.69]	5.34 [5.09–5.69]	5.14 [4.69–5.69]	0.4373
AUDIT Score	9 [0–10]	10 [9–12]	9 [0–9]	0.0091
OHI	1.66 [1.00–2.49]	1.00 [0.16–2.16]	1.66 [1.00–2.49]	0.1014
DMFT	2 [1–5]	1 [0–5]	2 [1–5]	0.2245
DPSI	2 [2–2]	2 [2–2]	2 [2–2]	0.2263

Note: NCOs: non-commissioned officers; %: percentage; NA: not applicable; IQR: inter quartile range; SBP: systolic blood pressure; DBP: diastolic blood pressure; BMI: body mass index; AUDIT: alcohol use disorders identification test; OHI: oral hygiene index; DMFT: decayed, missing and filled teeth index; DPSI: Dutch periodontal screening index; p*: Fisher exact test; p**: Mann Whitney test.

**Figure 1.**
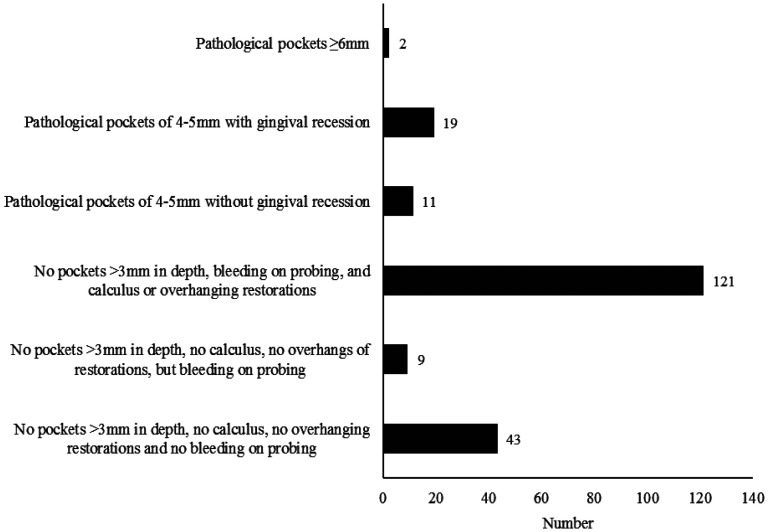
Participants and their clinical periodontal characteristics.

Concerning oral hygiene, 81 (39.5%) participants had good hygiene, 94 (45.9%) medium level of hygiene, and 30 (14.6%) poor oral hygiene. PD was present in 162 (79.0%) participants. The distribution of PD was not significantly different among officers and NCOs. The general characteristics of the sample are presented in Table 1 and Figure 1.

### 3.2. Relationship between oral health and cardiovascular risk profile

**Table 2.** Relationship between cardiovascular risk factors and periodontal diseases.

Cardiovascular risk factors	Gingivitis			Periodontitis		
	OR	95%CI	P	OR	95%CI	p
Smoking	0.88	[0.36–2.15]	0.8204	4.44	[1.73–11.43]	0.0031
Excessive alcohol consumption	1.06	[0.59–1.88]	0.8832	1.91	[0.83–4.36]	0.1693
Obesity or overweight	0.91	[0.51–1.64]	0.8824	1.13	[0.52–2.46]	0.8454
Hypertension	1.99	[0,85–4.68]	0.1188	0.49	[0.14–1.72]	0.4305
Diabetes	1.16	[0.10–12.97]	1.0000	2.76	[0.24–31.35]	0.4006

Note: OR: odd ratio; 95%CI: 95% confidence interval.

**Table 3.** Relationship between age, gender, periodontal parameters and high cardiovascular risk.

Variables	Univariate analyze			Multivariate analyze*		
	OR	95%CI	p	OR	95%CI	p
Age	1.11	[1.06–1.15]	0.0000	1.11	[1.06–1.16]	0.0000
Gender (M/W)	1.43	[0.40–5.09]	0.5771	-	-	-
DPSI	1.33	[0.95–1.87]	0.0992	-	-	-
Quality of oral hygiene						
Poor/Good	6.51	[1.97–21.52]	0.0021	3.96	[1.07–14.57]	0.0386
Medium/Good	2.88	[1.00–8.33]	0.0501	3.44	[1.11–10.66]	0.0322

Note: *: variables in the logistic regression model: age and quality of oral hygiene; OR: odd ratio; 95%CI: 95% confidence interval; M: men; W: women.

Regarding the relationship between oral health and cardiovascular risk factors smoking was associated with periodontitis (Odd Ratio: 4.44 [1.73–11.43], p = 0.0031).

The quality of hygiene was found to be associated with the cardiovascular risk profile regardless the age. Thus, participants with poor and medium oral hygiene quality had a high cardiovascular risk profile (Odd Ratio [95%: 3.96 [1.07–14.57], p = 0.0386 and 3.44 [1.11–10.66], p = 0.0322, respectively).

The results on the relationship between periodontal health and cardiovascular risk profile are presented in Tables 2 and 3.

## 4. Discussion

The objective of the current study was to assess the link between oral health of military from the Ngaoundéré garrison and their cardiovascular risk profile. Tobacco consumption was notably associated with periodontitis.

These results reinforce the idea that PD and CVD have common risk factors. Tobacco is a classic risk factor known for both PD and CVD. Likewise, several studies have highlighted the fact that poororal hygiene which is the main risk factor for PD increases the risk of CVD.

In addition of assessing the link between periodontal health and the cardiovascular risk profile of soldiers from the Ngaoundéré garrison, this study provides the first data on oral health of Cameroonian soldiers. Thus, regarding the distribution of PD in among the 205 participants included, the frequency of gingivitis was 64.4% and that of periodontitis, 15.6%. These results showed that the frequency of gingivitis and periodontitis was similar to the general population (62% and 15% respectively) in the Cameroon as reported by Essama et al. [8] in a multicenter study.

Tooth decay, can be considered as low since the median DFMT was equal to 2. This low level of tooth decay could be explained by the fact that only 14% of the participants had poor oral hygiene.

Regarding cardiovascular risk factors, we found that the distribution of behavioral risk factors seems to be higher than in the general Cameroonian population, smokers in the current study reprensented11.2% compared to 8.3% in the general population of Ngaoundéré [24]. On the other hand, participants were less affected by diabetes and hypertension; 1.4% diabetic against 5.8% [25]; and 16.10% hypertension, against 32.1% in the Cameroonian general population [26]. This lower affection by diabetes and hypertension could be explained by the fact that Cameroonian soldiers carry out regular physical activity compared to the general population. All military units in Cameroon have at least two compulsory days of collective sports per week.

A couple of limitations of the current study, could be the epidemiological tools used to determine the periodontal status of participants and their cardiovascular risk profile. In fact, regarding the cardiovascular risk profile, we opted for the summation of risk factors as a method of determining this risk profile because more economic and easier to implement. Indeed, the most widespread methods of determining cardiovascular risk use scores, but these scores were defined from studies carried out on non-African populations and their use on African people remains controversial. Also, the use of the cardiovascular risk prediction tables of the WHO and the International Society of Hypertension, but these are dedicated to subjects over 40 years. We therefore retained the summation of the risk factors already described in the literature [27]. The periodontal status assessment tool did not pose any problem because the DPSI is a validated index already widely used [28].

## 5. Conclusions

Association between some oral health status parameters and cardiovascular risk profile of military of the Ngaoundéré garrison has been highlighted. This call, military to correct certain lifestyle habits such as tobacco consumption and poor oral hygiene, which are harmful both to periodontal and cardiovascular health.
